# On Image Fusion of Ground Surface Vibration for Mapping and Locating Underground Pipeline Leakage: An Experimental Investigation

**DOI:** 10.3390/s20071896

**Published:** 2020-03-29

**Authors:** Shuan Yan, Hongyong Yuan, Yan Gao, Boao Jin, Jennifer M. Muggleton, Lizheng Deng

**Affiliations:** 1 Institute of Public Safety Research, Dept. of Engineering Physics, Tsinghua University, Beijing 100084, China; yan-s17@mails.tsinghua.edu.cn (S.Y.); hy-yuan@tsinghua.edu.cn (H.Y.); dlz17@mails.tsinghua.edu.cn (L.D.); 2 Key Laboratory of Noise and Vibration Research, Institute of Acoustics, Chinese Academy of Sciences, Beijing 100190, China; jinboao16@mails.ucas.ac.cn; 3 University of Chinese Academy of Sciences, Beijing 100049, China; 4 Institute of Sound and Vibration Research, University of Southampton, Highfield, Southampton SO17 1BJ UK; jmm@isvr.soton.ac.uk

**Keywords:** connected graph traversal, ground surface vibration, sensor array, pipeline leakage, leakage detection

## Abstract

This paper is concerned with imaging techniques for mapping and locating underground pipeline leakage. Ground surface vibrations induced by the propagating axisymmetric wave can be measured by an array of acoustic/vibration sensors, with the extraction of magnitude information used to determine the position of leak source. A method of connected graph traversal is incorporated into the vibroacoustic technique to obtain the spatial image with better accuracy compared to the conventional magnitude contour plot. Measurements are made on a dedicated cast iron water pipe by an array of seven triaxial geophones. The spectral characteristics of the propagation of leak noise signals from underground water pipes to the ground surface are reported. Furthermore, it is demonstrated that suspicious leakage areas can be readily identified by extracting and fusing the feature patterns at low frequencies where leak noise dominates. The results agree well with the real leakage position in the underground pipeline.

## 1. Introduction

As an important modern lifeline, the water supply network is closely connected to all aspects of our society, and the integrity of which provides essential services to production and residential life. Delayed and ineffective maintenance of the underground network may quickly trigger chain interruptions of public services [1]. However, the methods currently available to detect and repair the underground water supply network are prone to damage to other infrastructures such as road surface, imposing large impacts on the residential environment and public transport. It is estimated that billions of dollars are spent annually on maintaining these networks and other infrastructures in a country such as Australia [2], with the majority of the expense due to direct excavation damage. Therefore, to reduce the social, environmental, and economic consequences, methods for effective and accurate detection and location of pipeline leakage are urgently required [3].

Previous studies have shown that the vibroacoustics technique can be adopted to locate the leakage in buried pipelines through measurements using acoustic/vibration sensors either installed along the pipeline or on the ground surface due to the dispersion and radiation characteristics of leakage signals [4,5,6,7,8,9,10,11,12,13,14]. Moreover, the principle of interior detection is based on leak noise propagation in a tube: a microphone is mounted within a sphere that runs with the water flowing in the pipe [15]; when the sphere moves to the vicinity of the leakage point, the acoustic signal collected by the sensor will increase rapidly, resulting in larger signal amplitude when the distance between them gets shorter. Although the sensor is sensitive to leakage, it is difficult to control the movement of the sphere, and therefore difficult to accurately determine the leakage position. Alternatively, exterior detection methods have been developed by analyzing the signals measured on the pipe wall [16,17]. In these methods, signals are collected by two sensors located either side of a suspected leak, with the phase spectrum in the frequency domain (or time difference in the time domain) used to determine whether there is a leak or not and to locate the leak position. The disadvantages of this method are that (1) the leakage detection range is relatively low in the presence of background noise and (2) it is not applicable to the multi-leakage detection.

Listening devices have been used on the ground to pinpoint the leak position for a long time, which generally require a single detector composed of a transducer (for example a ground microphone), a signal processor, and an earphone [18]. A ground transducer collects the vibrational signals on the ground surface induced by leakage, which are then transformed into electrical signals for amplification and filtering through the signal processors before the leakage position can be determined by human perception. Albeit straightforward, this method greatly relies on human experience and large interference caused by human factors that may be involved. In theory, the leak noise, which is predominantly governed by the axisymmetric (n = 0) fluid-borne (s = 1) wave at lower frequencies, can radiate to the ground surface [19]. This wave has been studied in much of the previous work by Gao et al. [14,17,19]. It is a predominantly fluid-borne wave displaying approximately non-dispersive behavior at lower frequencies. Based on the radiation characteristics of the propagation s = 1 wave in the soil, the sensor array on the ground surface has been developed and used for pipeline location [20]. It has been suggested that the magnitude information is beneficial to the identification of a leak, as the leakage detection on the ground surface follows the same principle. In this circumstance, the leakage can be detected more accurately even in the occurrence of multiple leakage without resorting to the experience of the user. Imaging processing has shown its potential in combination with the vibroacoustic methods in the application of mapping and locating the pipeline leakage. Nevertheless, there are still a number of gaps in the existing techniques. Methods of connected graph and moment estimation are effective for the division of unconnected regions [21,22,23,24,25]. This indicates that attempting to analyze the contour distribution of the vibroacoustic signals by image fusion may lead to the improved image results for leakage detection.

In this paper, image fusion is exploited and incorporated into the vibroacoustic technique to map the ground surface vibration, thereby demonstrating a feasible approach for remote sensing and locating underground pipeline leakage by using a sensor array installed on the ground surface. Following the introduction of the method of connected graph traversal, a description of the experimental rig is given, along with the spectral characteristics of the propagation of leak noise. Further analysis on image fusion is carried out to show the promise of the proposed method for improving the accuracy of leak position over the conventional vibroacoustic method. To assist the reader for better understanding the rationale behind our method, in Appendix A, image fusion is applied to the ground surface vibration measurements for the pipe location reported in [20], confirming the improved performance of the vibroacoustic technique.

## 2. Methodology

Before considering image fusion in combination with the vibroacoustic technique for mapping and locating underground pipeline leakage, the concepts of connected subgraphs and moment estimation are briefly introduced in this section.

### 2.1. Search of Connected Subgraphs

**Figure 1:**
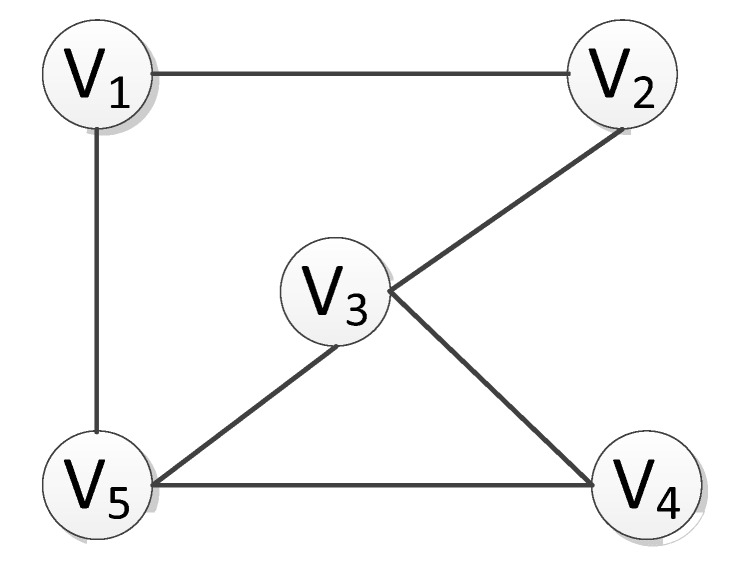
Schematic diagram showing connected graph with loop.

**Figure 2:**
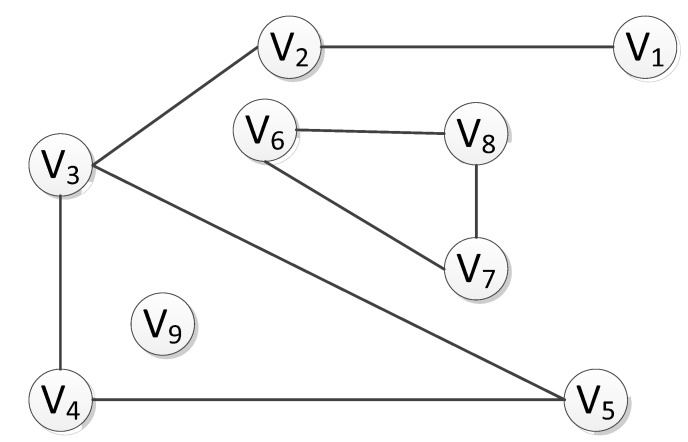
Schematic diagram showing multiple connected subgraphs and independent nodes with loops.

In an undirected graph G, if there is a path <V_1_, V_2_> from vertex V_1_ to vertex V_2_, and then V_1_ and V_2_ are considered to be connected. If any two vertices V_i_ and V_j_ (V_i_, V_j_∈Vertex) are connected, then the undirected graph G is a connected graph. As illustrated in Figure 1, the undirected graph is a connected graph, whereas the undirected graph in Figure 2 is a non-connected graph, despite the existence of three connected components. Here, the terminology “connected component” [21,24,25] refers to the maximum connected subgraph in an undirected graph.

The path edge <V_1_, V_2_> in the undirected graph G represents the position and adjacency between vertices. In a directed graph, however, this adjacency represents a path, and the path edges <V_1_, V_2_> and <V_2_, V_1_> are different paths, representing different ways of linkage, thereby leading to different search paths. In the undirected graph, however, the path edge <V_1_, V_2_> is a relative concept, because there is no direction defined in the undirected graph. Therefore, this adjacent edge can point either from V_1_ to V_2_, or from V_2_ to V_1_. By definition, with respect to the logical structure of the whole graph, there is no total order relationship between the vertices of an undirected graph. Thus, it is impossible to arrange the vertices in the graph into a unique and fixed linear sequence, in that each vertex can be treated as the starting vertex. When sorting the points adjacent to a particular vertex, there may be multiple sorting results without a special order of sequence for the nodes in the sorting.

In the process of the traversing graph, we start from one vertex to visit the rest of the vertices of the graph, to ensure that each vertex is visited only once. In this paper, to better search the connected subgraph, each node is allowed to pass through many times. The algorithm of the connected graph traversal provides the basis for solving the connectivity problem of graph. It is noted that because any vertex of a graph may be adjacent to other vertices, after accessing a vertex, the algorithm may search along a path and return to the vertex again. For example, the larger connected component as illustrated in Figure 2 can be accessed to V_3_ along the edge (V_5_, V_3_) after accessing V_1_, V_2_, V_3_, V_4_, and V_5_ because of the presence of loop. Generally, there are two paths of graph traversal including the depth-first search and the breadth-first search, which are both applicable to undirected graphs. 

### 2.2. Moment Estimation

In the maximum connected subgraph and the feature region containing each connected component, all nodes are calculated as matrix elements. The method of moment estimation [22,23] can be used to calculate the origin moment and central moment of the whole matrix, based on which the centroid of the feature region can be derived.

For the feature region f (x, y) of M * N, the (p + q)-th order mixed origin moment is given by

(1) $$m_{\mathrm{pq}}=\overset{M}{\underset{x=1}{\sum }}\overset{N}{\underset{y=1}{\sum }}x^{p}y^{q}f\left(x,y\right)$$

The central moment of the (p + q)-th order mixing is obtained by

(2) $$\mu_{\mathrm{pq}}=\overset{M}{\underset{x=1}{\sum }}\overset{N}{\underset{y=1}{\sum }}{\left(x-x_{0}\right)}^{p}{\left(y-y_{0}\right)}^{q}f\left(x,y\right)$$

where x_0_ and y_0_ are the centroid coordinates selected in the current round of calculation. The normalized central moment of the (p + q)-th order is given by

(3) $$\eta_{\mathrm{pq}}=\frac{\mu_{\mathrm{pq}}}{\mu_{00}^{r}}$$

Based on the first-order origin moment, the coordinates of the centroid of each continuous region can be solved as

(4) $$\bar{x}=\frac{m_{10}}{m_{00}}$$

(5) $$\bar{y}=\frac{m_{01}}{m_{00}}$$

## 3. Initial Experiments on Test Rig

### 3.1. Experimental Set-Up

**Figure 3:**
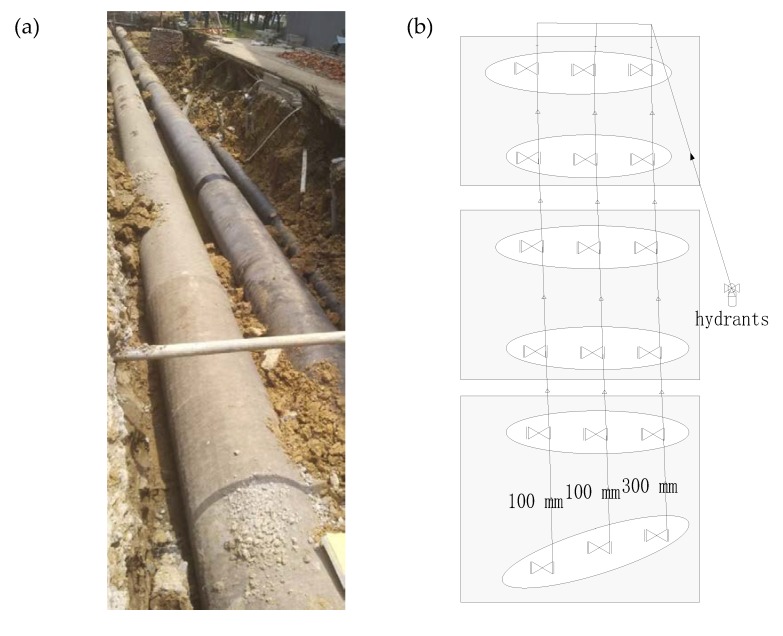
Pipe rig layout: (**a**) photograph showing the test rig under construction; (**b**) schematic of three test pipes.

The aim of the experimental measurements is to explore the use of ground surface vibration signals for mapping and locating the leakage from buried pipeline. Experiments were carried out at the Hefei Institute of Public Security, Tsinghua University. Pipes with different calibers were laid out on the integrated pipeline platform of the institute. The pipe rig layout under construction is shown in Figure 3. It consisted of five pipes in parallel, three of which as shown in Figure 3a were chosen to be tested, including two cast iron and one PE water pipes with diameters of 300 mm, 100 mm, and 100 mm from right to left in Figure 3b, respectively. The total length of each pipe was 91 m. 

**Figure 4:**
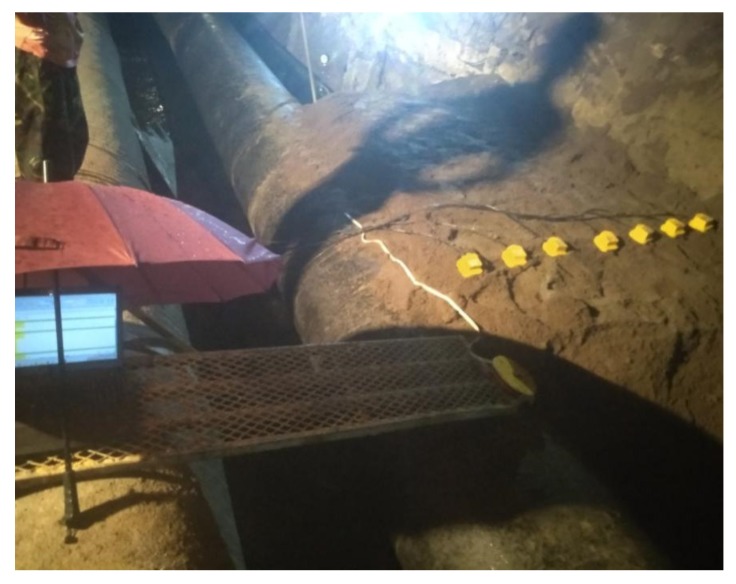
Photograph showing the measurement arrangement for ground surface vibration.

In the experiments, slots of 1 mm × 10 mm were opened as simulated leakage sources at different positions on the pipe walls. Leak sources were covered by soil of different depths, and mechanical equipment was used to compact the soil. As shown in Figure 4, in each measurement, an array of 7 SM-24 tri-axial geophones with spacing of 0.25 m were positioned on the ground surface aligned perpendicular to the pipelines. This led to the vibrational velocities measured in three axial directions representing vertical measurement, horizontal measurement in line with the pipe, and horizontal measurement perpendicular to the pipeline. It is found that the data collected in line with the pipeline is the most informative and thereby being selected in the analysis for determination of the leakage position. The data was collected for a duration of 60 s at a sampling rate of 8192 Hz, by using the acquisition system of Type-MKII produced by BBM PAK. Experiments were carried out at night to minimize the influence of environmental noise. 

### 3.2. Determination of the Frequency Range for Leakage Detection

Previous investigations [20] have shown that the choice of frequency range is crucially important to accurately map and locate the pipeline, suggesting that the dominant s = 1 wave can be effectively radiated into the soil in the frequency range of interest. It must be kept in mind that the s = 1 wave is also the main energy carrier due to water leakage in underground piping system. Therefore, for the vibroacoustic technique to be effective for the leakage location, the frequency range needs to be carefully determined prior to the imaging process in the next section. In this subsection, the effects of the measurement position and burial depth (calculated above the pipe) are studied as follows. 

(1) Effect of the horizontal distance from the leakage source

**Figure 5:**
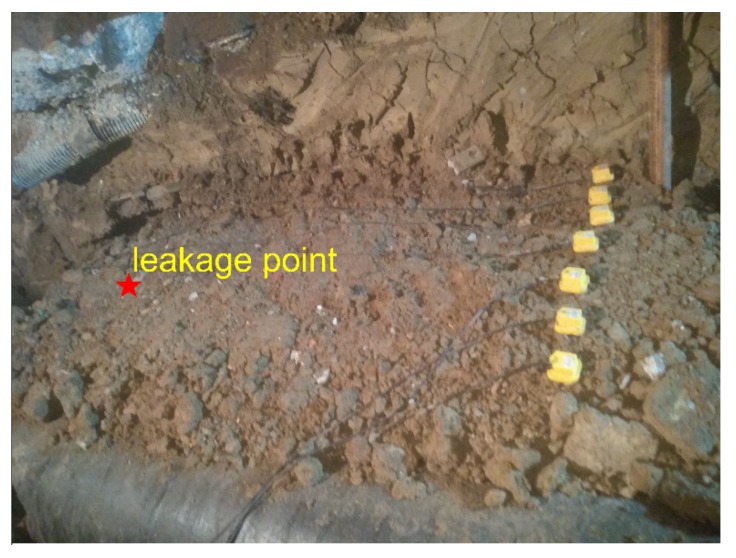
Ground surface vibration measurement using the sensor array at the distance of 1.5 m from the leak.

**Figure 6:**
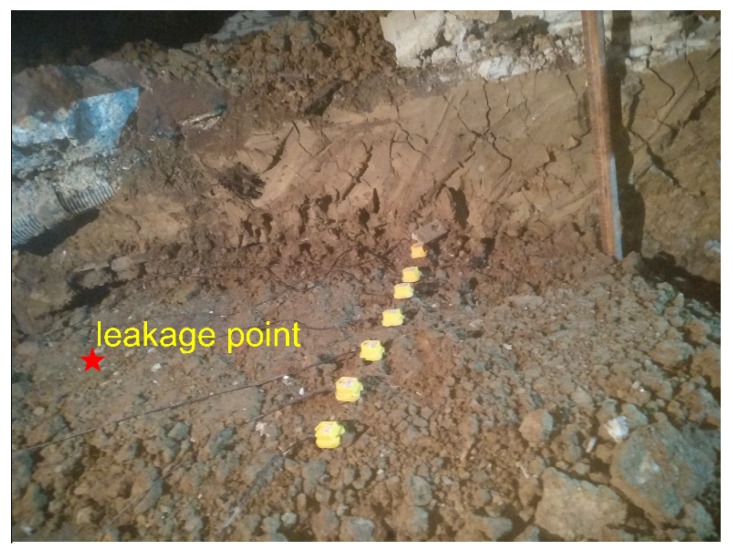
Ground surface vibration measurement using the sensor array at the distance of 1 m from the leak.

**Figure 7:**
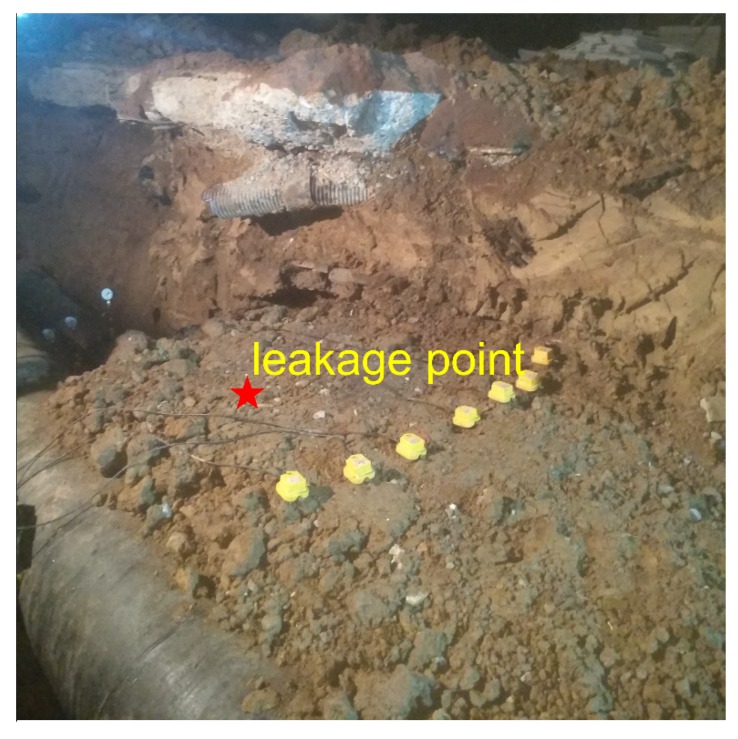
Ground surface vibration measurement using the sensor array at the distance of 0.5 m from the leak.

Three sets of measurements were conducted at three different distances using the sensor array to collect the vibrational signals on the ground surface induced by the same leak source from the cast iron pipe (with the diameter of 300 mm). The pipelines were buried in clay soil at a depth of 1 m, and the water pressure in the pipe was 0.2 MPa. The sensor array was allocated at the distances of 1.5 m, 1 m, and 0.5 m form the leak, as illustrated in Figure 5, Figure 6 and Figure 7.

**Figure 8:**
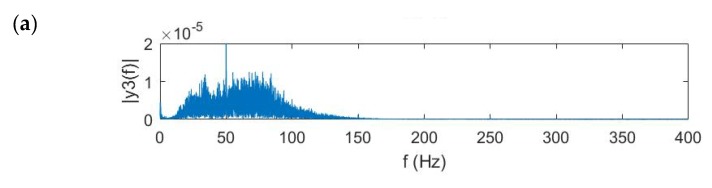

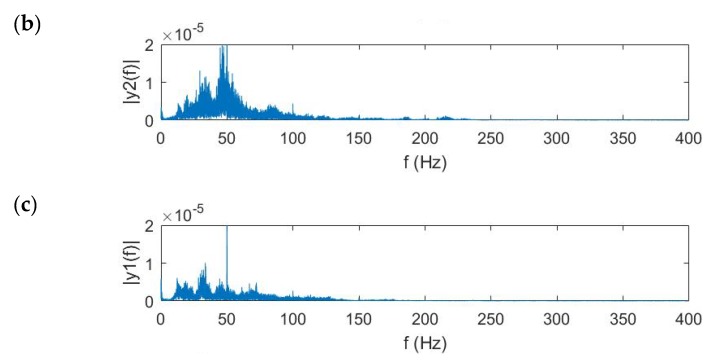
Frequency domain vibrational velocity on the ground measured at the distances from the leak of (**a**) 0.5 m, (**b**) 1 m, and (**c**) 1.5 m.

Figure 8 shows the frequency response of ground surface vibration signals measured by the middle sensor in the array at different distances away from the leak. It is apparent, the magnitude of the vibrational signal increases when the sensor array moves towards the leak, in particular the magnitude at higher frequencies. This demonstrates that the soil heavily attenuates the leak noise signal, especially at higher frequencies. The frequency range of the vibrational signal is thus chosen based on its frequency response for further imaging processing. For the distances between the sensor array and leak source of 1.5 m and 1 m, the frequency range is set to be 20 Hz to 100 Hz; for the distance of 0.5 m, the frequency range is set to be 20 Hz to 130 Hz.

(2) Effect of the burial depth

To investigate the effect of the burial depth on the frequency range due to leakage, the vibrational velocity was measured on the ground surface just above the leak source on the same cast iron pipe as mentioned above in the measurements. The buried depths were set to be 0.5 m, 1 m, and 1.5 m, and the water pressure in the pipe remained as 0.2 MPa.

**Figure 9:**
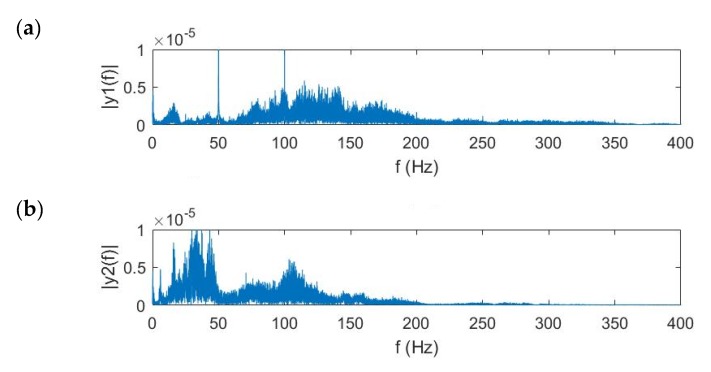

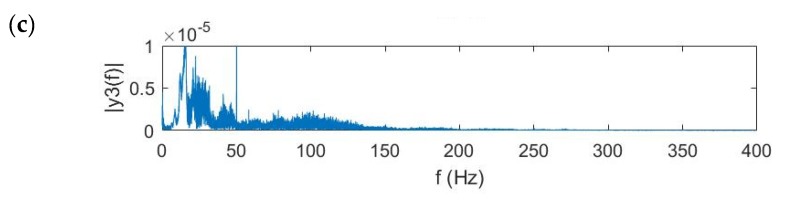
Frequency domain vibrational velocity on the ground measured at the burial depth of (**a**) 0.5 m, (**b**) 1 m, and (**c**) 1.5 m.

Figure 9 shows the frequency response of the measured ground surface vibration. It can be seen that for the pipe buried deeper, the frequency range where the leak noise is dominant becomes narrower. For the burial depth of 0.5 m, the effective bandwidth is 20–240 Hz, with the central component at ~130 Hz. For the burial depth of 1 m, the frequency range becomes 20 to 160 Hz, with the central component at ~40 Hz. Note that the peaks at 50 Hz and 100 Hz are caused by power frequency disturbance. Because of the interference of the power frequency, several peaks can be found at integral times of 50 Hz. For the burial depth of 1.5 m, the effective bandwidth reduces to 20–100 Hz, with the central component at ~30 Hz. It has been demonstrated that the burial depth has large influence on the magnitude of vibrational signals included by leakage at higher frequencies.

## 4. Image Analysis

**Figure 10:**
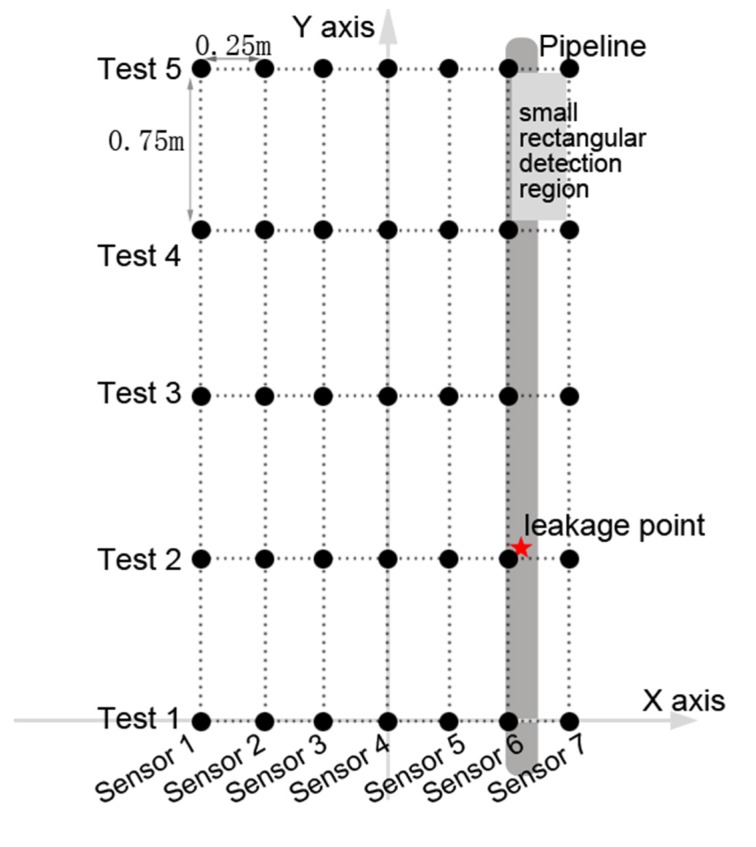
Grid of measurement points.

In the experiments, an array of seven geophones (as mentioned in Section 3) was positioned on the ground surface with the one of the sensors directly above the pipeline. Five sets of measurements were made over a rectangular grid of measurement points up to 3 m along the pipeline and 0.75 m either side. The grid spacing was set at 0.25 m and 0.75 m in the x- and y-directions, respectively, leading to a total of 7x5 measurement points, as shown in Figure 10. The simulated leak was located near (0.5, 0.75) as marked by a red star in the figure. It must be pointed out that the experimental rig was built to verify the effectiveness of the proposed image fusion method. Thus, the data collected in the measurements is analyzed to reveal the suspected leakage position in comparison with the actual leakage area. As suggested in the preceding section, the soil has a great influence on the frequency range of the ground surface vibration signals due to leakage. A further check on the frequency domain vibrational velocities measured shows that the magnitude levels are significant between 60 Hz and 100 Hz for all dataset. Thus, the image analysis is now conducted on the data set spaced every 10 Hz in this frequency range. 

**Figure 11:**
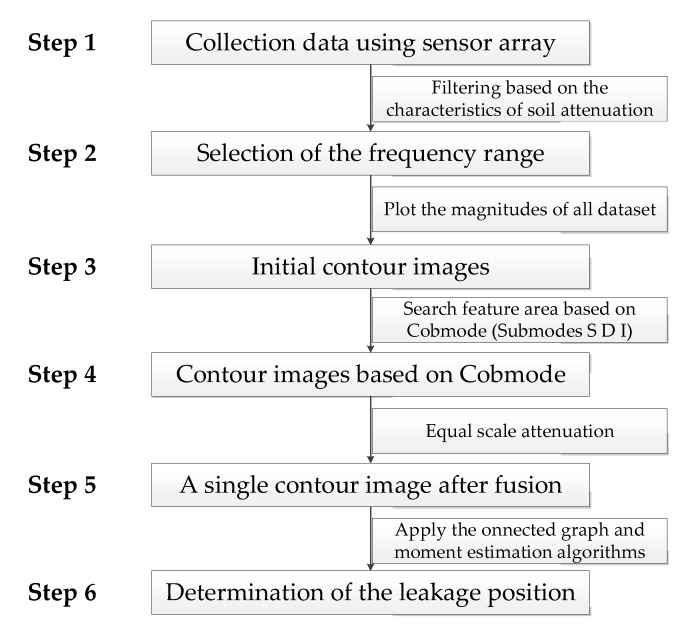
Steps for the mapping and locating the pipe leakage based on ground surface vibration measurements.

Steps for the mapping and locating the pipe leakage based on ground surface vibration measurements are illustrated in Figure 11. Ground surface measurements are first made by using sensor array in Step 1. Based on the attenuation characteristics described in Section 2, filtering operation is conducted on the data to determine the frequency range for the image analysis (being 60–100 Hz for the test data here). The remaining steps for the imaging process will be discussed in the following subsections.

### 4.1. Contour Image Analysis

**Figure 12:**
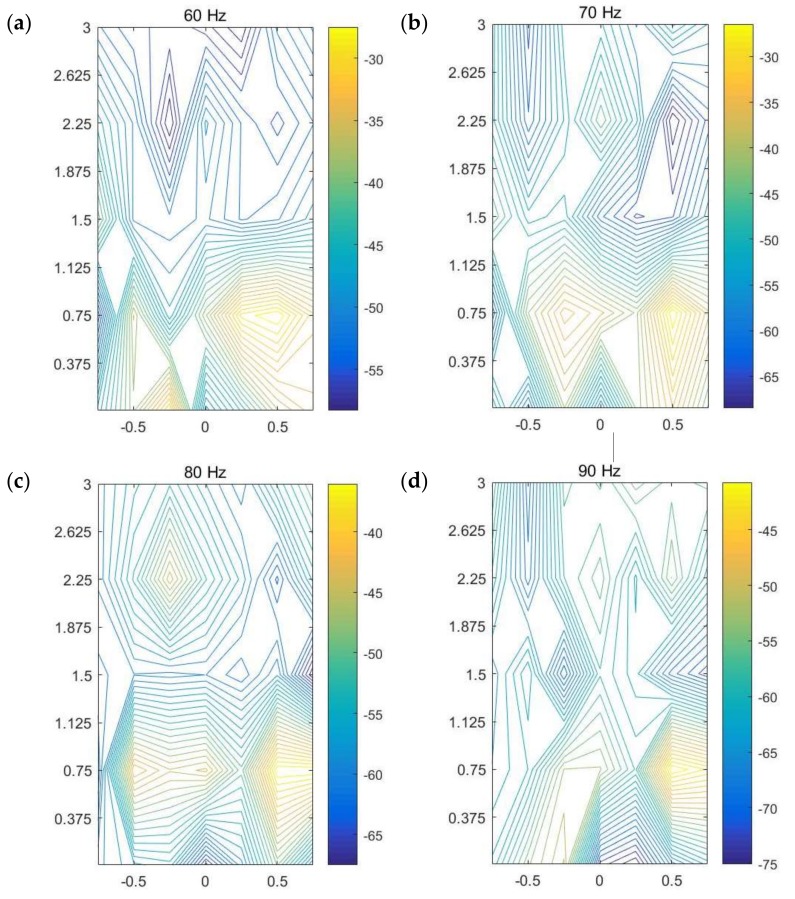

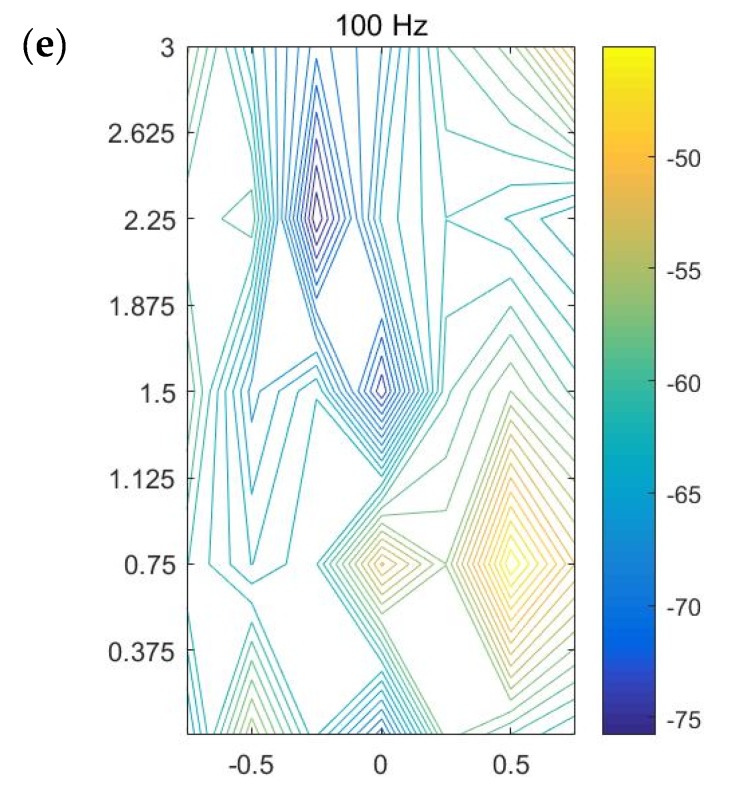
Magnitude contour images of ground surface vibration measurements at five frequencies.

To accurately identify the leakage area, an image pattern recognition algorithm in Step 4 is proposed and developed here for analyzing the combination of the initial magnitude contour plots of the ground surface vibration measurements in Step 3. In the contour image, the x- and y-coordinates match with the x- and y-axes as shown in Figure 10, with the origin set at the position of the middle sensor in Test 1. The reader is referred to the work in [20] for detailed information on the description of magnitude contour image using all dataset (not repeated here). Figure 12 shows the contour plots at five frequencies. The energy bar in each figure determines the range of the energy distribution, based on which different weights are applied to individual images according to the difference between the upper and lower bounds of the energy bar. 

Three submodes are now identified from the contour images at different frequencies plotted in Figure 12, with their combination used in mathematical modeling for accurately detecting and locating the leakage area. This combination of sub-modes is termed “CobMode”, and the three submodes include the following

(1) Submode S (Surround) is a wrap mode, for which gradual variation of gradient is present around the leakage point, leading to a uniform closed-loop. Moreover, more complete envelopes can be obtained between the closed-loop contours seen as a more distinct mode of gradual variation of the “maximum–minimum” value close to leakage.

(2) Submode D (density) represents features of contour density and gradient change. Both the density and the absolute value of gradient of contour are considerably large around the leak source, which attenuate from the leak source signal outwards.

(3) Submode I (intensity) is obtained based on the magnitude of each contour line or the color depth value of the image. In general, an actual leak leads to ground surface vibration with great intensity just above it, thus a comparison of the magnitude values is used to judge whether there is a leak from the contour map. 

**Figure 13:**
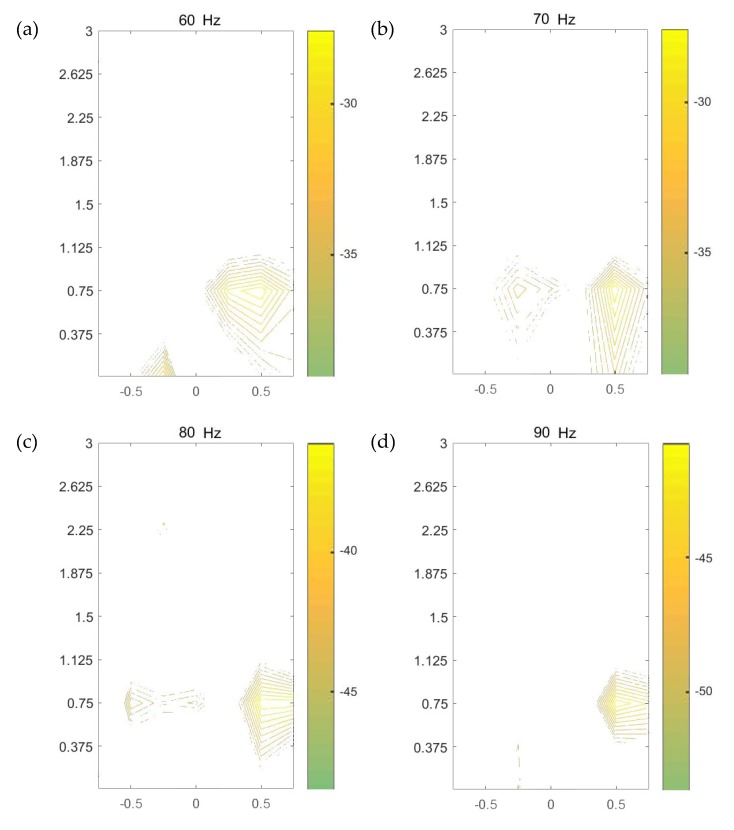

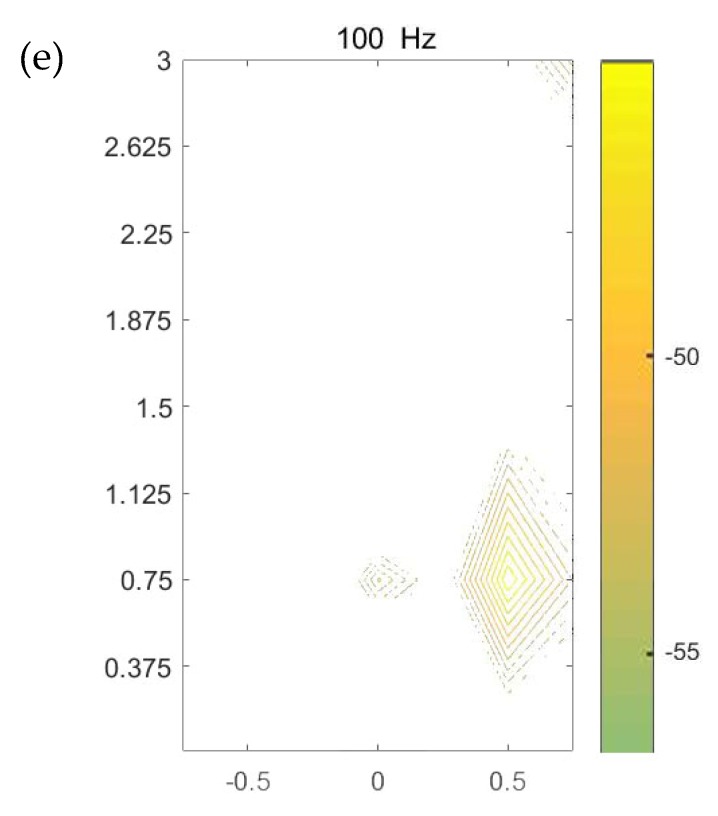
Contour image based on CobMode for Figure 12.

Consider the contour line corresponding to the path formed during the movement of each sensor in the array. Based on the distribution patterns of the three submodes in the contour lines of a target region and the variation of its adjacent region, the three submodes can be identified and combined as plotted in Figure 13. The distributions of the submodes (S, D, and I) can be observed in the figures. In each contour map at an individual frequency, the region where the three submodes are most highly concentrated and coupled is selected as the possible position corresponding to the leakage area. In most cases, the areas where the energy is relatively strong represent those where the three modes are concentrated. Comparing the mapping images at all frequencies considered as shown in Figure 13, it is clear that the area near (0.5, 0.75) is highlighted in each figure, implying a suspected leakage area directly below it. The prediction is in good agreement with the actual leakage position in buried water pipes at the test site.

### 4.2. Image Fusion

Possible leakage areas are highlighted at different frequencies in Figure 12. It is difficult to infer the suspicious leakage position using a single image, so multi-frequency data needs to be used for further analysis. A multi-image fusion algorithm in Step 5 is proposed based on the algorithm of pattern extraction, the method of maximum variance between classes, and the algorithm of image superposition, and applied to the plots in Step 4 to offer a framework for automatic determination of the most likely leakage position. Each contour image is first converted to an image file in the “.png” format. Based on the intensity corresponding to the top-down color of the energy bar, the areas with more concentrated large intensity are extracted from the image to construct another image which only contains the suspected leakage areas with the relatively weak signals in other areas filtered out. In the image extracted, the original relationship is preserved between the intensity and color value. The image at each frequency is converted to a gray-scale image with different depths. The upper part of the energy bar, such as yellow and red, has larger intensity, corresponding to larger color depth in the gray image, whereas the part with smaller intensity displays lighter color in the grayscale image. 

**Figure 14:**
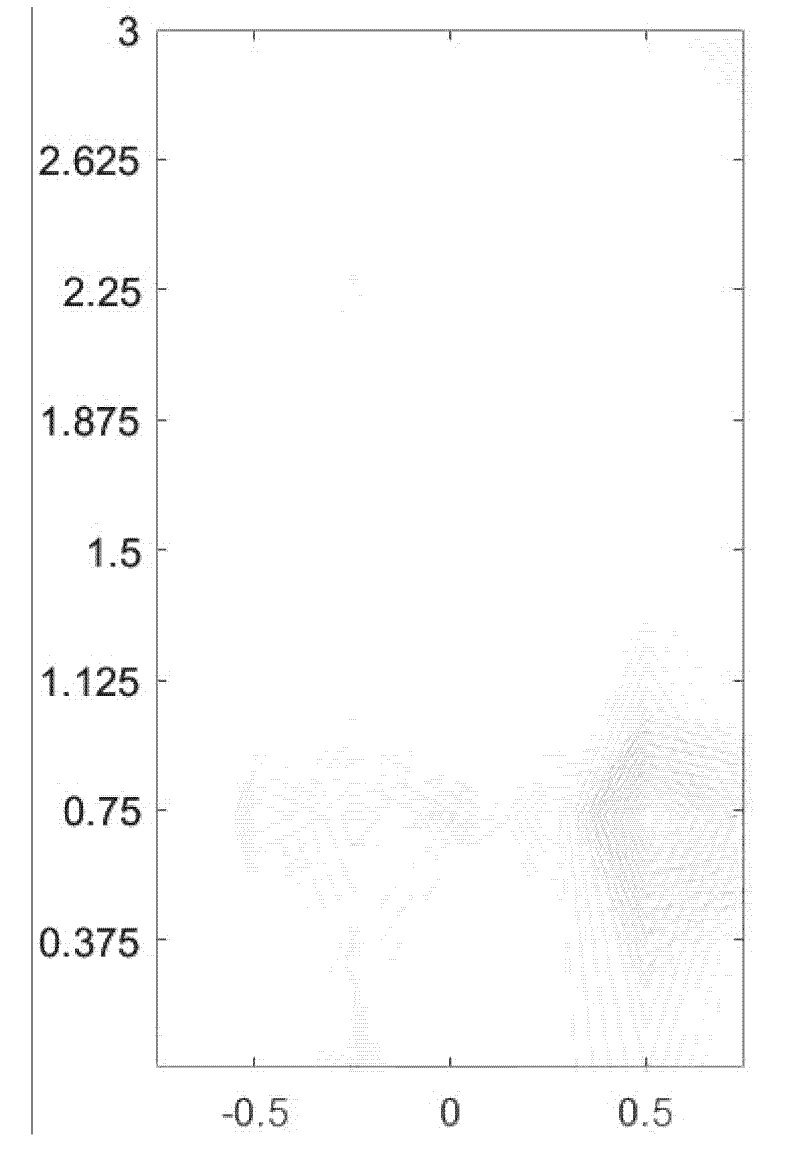
Grayscale image after fusion.

Image fusion is subsequently applied to the image sequence in Figure 13 to lead to a single image as plotted in Figure 14, according to the original coordinates in each grayscale image with the features being attenuated. The grayscale image, in contrast to the color image, is more conductive to the calculation of moment estimation. To avoid the overflow of color value, the fused image needs to be attenuated. The level of attenuation is determined by the number of image sequences and the intensities in Figure 13, thus ensuring that the feature extracted from each image can reflect the same color depth corresponding to the same intensity in the fusion image. The energy bars in the processed images coincide with those in Figure 12. As a result, the fused image is still a grayscale image with different color depths, which possess different fusion weights to the image sequence according to the number and upper and lower bounds of amplitude.

In the grayscale image, the method of maximum interclass variance is used to extract the limited edge. Through edge detection, the weaker area in Figure 13 becomes even weaker and is eliminated after the processing of fusion attenuation, whereas the areas with stronger features are retained. The method of maximum variance between classes is an adaptive method to determine the threshold, which divides the image into two parts including background and feature, according to the gray level of the image. The variance between the background and features reflects the difference between the two parts of the image. Therefore, the segmentation with the largest variance between classes corresponds to the misclassification with the minimum probability.

After the aforementioned processing, the pattern in the fusion image is displayed as many points and line segments. These line segments constitute many undirected graphs, including unconnected graphs, connected graphs, and connected subgraphs. By searching each connected subgraph of undirected graph as discussed in Section 2.1, the most likely leakage position can be readily identified as follows: (1) search the region based on the features of connected subgraph; (2) find all of the connected graphs in the fusion image; (3) compare and sort all the connected graphs based on the number of connected components; and (4) find the largest connected subgraph. If the image is not clear enough, the filling operation is suggested to quantify the surrounding area of the suspected leakage point. This can be achieved by filling the closed connected components with a specific color so as to compare the size of the smaller connected components.

**Figure 15:**
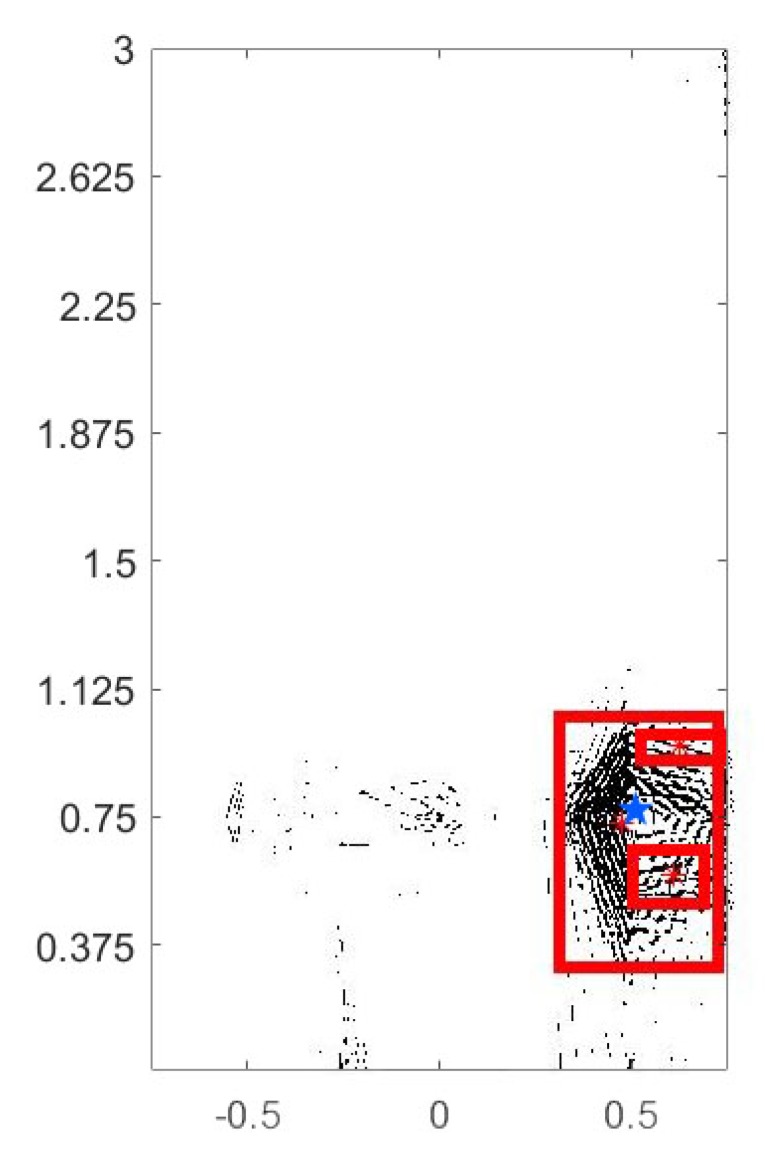
Determination of the suspected leakage area and leakage position.

For the fused image in Figure 15, the connected subgraph is used to divide multiple analysis areas, and then the energy distribution of the target area is analyzed. The discrete edges or points in Figure 14 and Figure 15 are composed of similar connected components as discussed in Section 2.1. The method of connected graph traversal is used to search not only the edges or nodes covered by connected subgraphs, but also the discrete, independently connected subgraphs or nodes surrounded by connected subgraphs. These scattered connected subgraphs and nodes together constitute the energy distribution map of the suspected leakage area. The existence of discontinuous points or edges in the fusion image after the final feature extraction does not suggest that the energy does not exist or is discontinuous at that point. The discontinuity may be caused by filtering out the weak signal edge in the earlier feature extraction between Figure 12 and Figure 14, or the signal is largely attenuated owing to the low-pass filtering characteristics of the soil medium between the source and sensors. Figure 15 show the maximum three suspicious leakage areas and their leakage points with red stars drawn in the fusion image, and the actual leakage position is marked with a blue star. The three suspected leakage areas are the three connected subgraphs with the most connected components within all connected graphs. For the fused image, the moment estimation method is used to solve the centroid in the divided region to obtain the accurate leakage location. The exact coordinate position can be obtained by picking up the coordinates of the leakage point. The central leakage point is indicated by red asterisk in the figure. 

## 5. Conclusions

In this paper, image fusion algorithms have been proposed and incorporated into the contour images obtained using vibroacoustic technique for mapping and locating the underground pipe leakage. Experimental investigations have been conducted on a dedicated cast iron water pipe, and ground surface vibration measurements have been made using an array of geophones. For the selected frequency range where the leakage signals dominate, a single contour image has been obtained by combining the image sequence at different frequencies. A method of connected graph traversal has been introduced to reveal the possible leakage area and hence leakage position. Experimental results using the method proposed in this paper have shown good agreement with the actual leakage area. It offers a potential improvement over the conventional vibroacoustic technique for automatically mapping and locating the suspected leakage area, which is beneficial to the practical application. In addition, this method greatly reduces the error caused by the subjective judgment and operator experience.

## Figures and Tables

**Figure A1:**
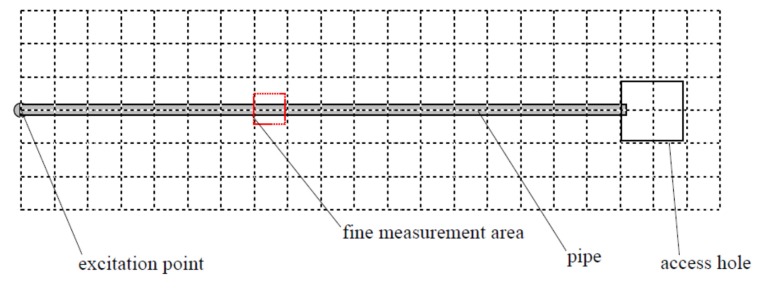
Grid of measurement points in [20].

**Figure A2:**
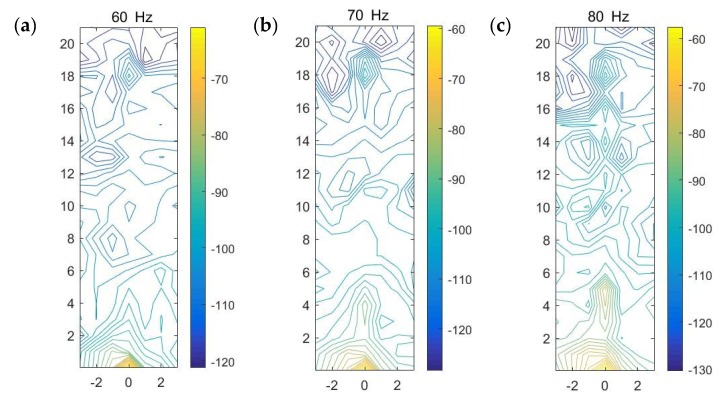

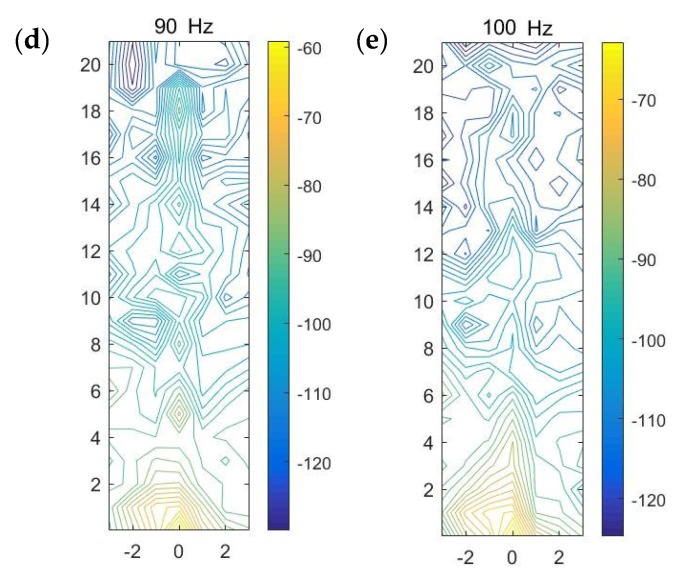
Magnitude contour image of ground surface vibration measurements at different frequencies.

**Figure A3:**
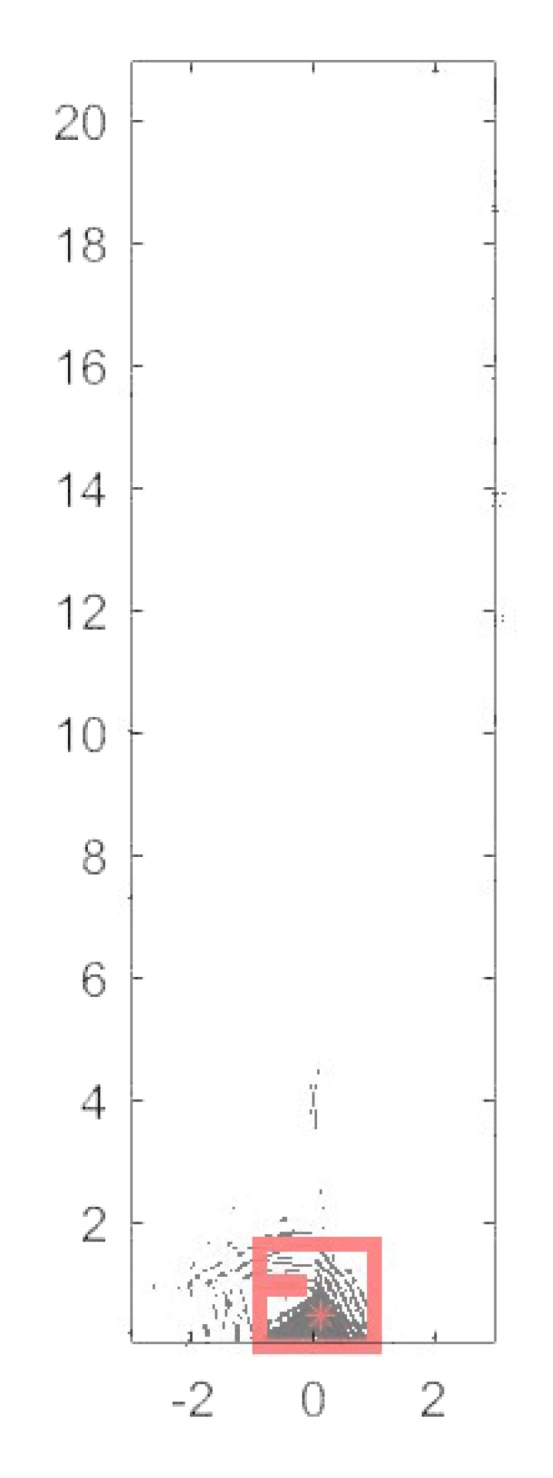
Determination of the excitation point after image fusion.

## Appendix A. Imaging Fusion on the Application of Pipe Location

The currently available vibroacoustic method for pipe location follows the same principle as the one for water leakage detection and location based on the ground surface measurements [20]. Although pipe location is not the main objective in this paper, it is, however, noteworthy that better performance may be achieved when adopting the proposed image fusion method for this application, and further confirming the improvements over the conventional vibroacoustic method. In this appendix, pipe location from ground surface measurements reported in [20] is analyzed. The detailed test procedures and frequency analysis are not simply repeated. The reader is referred to [20] for more information. The grid measurement points are shown in Figure A1. Analysis of the all dataset leads to the magnitude contour plots as plotted in Figure A2 at five frequencies. Instead of leakage position, the excitation point is found in the origin of the contour plots as suggested in [20]. Following the steps for image fusion proposed in the paper, a contour image is eventually obtained and plotted in Figure A3, with the excitation point clearly can be deduced. This implies that the image fusion algorithms are suited to pipe location.
